# Un/familiar connections: on the relevance of a sociology of personal life for exploring egg and sperm donation

**DOI:** 10.1111/1467-9566.12862

**Published:** 2019-02-22

**Authors:** Petra Nordqvist

**Affiliations:** ^1^ Department of Sociology University of Manchester Manchester UK

**Keywords:** egg donors, sperm donors, genetic connectedness, personal life, relationality, intimacy

## Abstract

In recent decades, reproductive medicine has become a widespread global phenomenon. Within the field, donor conception, and the use of donated eggs, sperm or embryos from a third party, plays a key role. Despite the importance of those individuals who donate, there has been scant research exploring their experiences. Seeking to contribute to the growing, albeit still small, body of research on donors, this paper advocates bringing the process of donating into dialogue with a sociology of personal life. It suggests that important new insights about the donor experience can be achieved by utilising such a theoretical perspective. The paper applies a broad framework of a sociology of personal life to demonstrate that the decision to donate reverberates within donors’ everyday lives and relationships, and explores, primarily theoretically, how it is that acts of donation bring such issues into play. To this end, the paper examines in detail three ways in which donating interacts with dimensions that are integral to personal life: “living” genetic connectedness, relationality and the intimate body. Ultimately, the paper suggests that a sociology of personal life shows light on new, unexplored questions for this field that demand greater scholarly attention.

## Introduction

Since the birth of Louise Brown in 1978, conceived through in vitro fertilisation (IVF) in Oldham, United Kingdom, reproductive medicine has become a global phenomenon, in terms of provision, uptake and experience (Inhorn 2015). Donor conception, or the utilising of donated eggs, sperm or embryos from a third party in order to conceive, has played a major part in this development. However, despite the crucial role of the men and women who choose to donate, research into the experiences of providers of gametes, as opposed to the recipients, remains surprisingly sparse (van den Broeck *et al*. 2013). This study seeks to contribute to a fledgling body of research (outlined below) that explores the experiences of people who donate eggs and sperm by suggesting that vital insights can be gained through the use of various tools and perspectives provided by a sociology of personal life.

A sociology of personal life emphasises the need to explore the prevailing importance of connectedness in people's lives, and is perhaps most clearly associated with Carol Smart's book *Personal Life* (2007). According to Smart (2007: 28), “‘the personal’ designates an area of life which impacts closely on people and means much to them.” The strength of this perspective is that it provides tools with which to investigate personal life as fundamentally relational, without being prescriptive about where meaningful forms of connectedness occur. Going beyond traditional boundaries such as “sociology of the family” or “sociology of work,” the sociology of personal life reveals how life experiences of connectedness emerge across a range of contexts and relationships, including, but not limited to, the family (e.g. May 2013). As such, this approach lends itself to a diverse range of analyses, e.g. exploring a sense of kinship in human–animal bonds (Charles 2014) and tango dancing (Törnqvist 2018). The sociology of personal life emerged, in part, from a critical engagement with social theory and its growing emphasis on individualisation over the past couple of decades (e.g. Bauman 2003, Beck and Beck‐Gernsheim 2002). In doing so, it builds on and extends the seminal work on the changing role of the family in the UK associated with, e.g. Finch (1985), Finch and Mason (1993, 2000) and Morgan (1996), a body of work whose roots can be traced back to Mead's (1934) proposition of the self as always constituted in relationship to others. At its core, the sociology of personal life recognises individual life as deeply constituted in and through its relational context. Thus, it enables a particular field of vision for the researcher: “an awareness of connection, relationship, reciprocal emotion, entwinement, memory [and] history” (Smart 2007: 189).

What might a sociology of personal life bring to the field of egg and sperm donation research? At present, the tendency is to study egg and sperm donors as isolated individual operators, as though “unattached.” Like everyone else, however, they come with connections of their own: they are someone's partner, child, mother, father, friend and so on. Sociologically speaking, decisions about donating are likely to raise significant questions for donors in terms of their own personal lives, including in relation to the people to whom they are themselves connected. To date, we have a very limited knowledge about what these questions may be, and how they may be resolved. We do know, however, that the field itself has developed in recent years, in ways that bring these issues to the fore. Whereas the donation process used to be managed through secrecy (Nordqvist 2014a), there has been a general shift towards openness in a growing number of countries, such as the UK, Sweden, parts of Australia, New Zealand and Switzerland. The anonymous donor relationship has, thus, in these locations at least, transformed into a relationship that is known and transparent (Klotz 2014). Looking at the UK in particular, this was most clearly marked by the removal of donor anonymity through the Human Fertilisation and Embryology Authority (HFEA) (Disclosure of Donor Information) Regulations 2004. Donors who donate in the UK after 1 April 2005 do so in the knowledge that any child born as a result of their donation can seek contact when they turn 18, a scenario that will begin to take effect in 2023.

Although evidence suggests that the act of donating can have long‐term consequences for anonymous donors (e.g. Konrad 2005, Shaw 2007), this new emphasis on transparency arguably represents a step change in terms of what these consequences might mean in respect of donors’ personal lives: they have made donors more “visible” within the process as a whole, but they have also, crucially, made donors contactable. Keeping the act of donation a secret has become less of an option for donors: a young adult might make contact through an established process, such as that offered by the HFEA UK, or the Victorian Assisted Reproductive Treatment Authority in Victoria, Australia. Such contact might prompt a range of questions or require explanation, not least to partners, parents or donors' own children. The move towards transparency thus means that donors are now likely to need to consider how best to manage the possibility of being contacted by their donor‐conceived offspring, in light of how the latter might in turn impact on their own lives and relationships. If donors have shared knowledge about having donated unevenly in their families, and evidence suggests that this is the case (see below), sensitivities are likely to have grown and become issues of negotiation and management between different family members; pockets of secrets may have developed (Nordqvist 2014b). As I shall go on to suggest, this means that contemporary egg and sperm donation likely evokes questions not just for donors, but also for wider family networks, as well as established ways of relating within such networks.

This paper is concerned with providing a way of understanding how donating raises and interacts with issues that are central to personal life. My principal aim is to show *that* the decision to donate resonates within donors’ everyday relationships and family lives, and to explore, primarily theoretically, *how* the act of donating generates issues of this nature. In doing so, the paper will provide an argument for the relevance of applying a personal life perspective in research with egg and sperm donors. In order to highlight how personal life comes to matter in this context, I explore three examples of connectedness brought to life in donating: “living” genetic connectedness, relationality and the intimate body. This list is not meant to be exhaustive; rather, the examples serve as illustrative instances that underpin my wider argument. I conclude by suggesting that a new research agenda is necessary to capture those aspects that are thrown into relief by the perspective of personal life**.**

Although my argument is explorative and theoretical in its nature, I shall at times draw on illustrative examples from recent studies in the field of reproductive donation. Partly, I draw on the existing, albeit limited, body of research into donors’ experiences. Existing studies, predominantly located in Australia, Denmark, New Zealand, the UK and the US, hint at the relevance of donors’ own personal lives within the donor experience (e.g. Daniels *et al*. 2012, Goedeke *et al*. 2015, Mohr 2016, 2018), yet without having used this as a specific theoretical lens. As a result, these studies remain somewhat limited in their ability to illustrate the relevance of personal life in any great detail. To mitigate this problem, I also draw on two of my own recent studies, exploring the experiences of recipient parents and grandparents of donor egg, sperm and embryos in England and Wales. This includes, first, my 2006–2009 Conceiving Together (CT) study, which explored lesbian couples’ experiences of becoming parents through donor insemination, and second, the 2010–2013 Relative Strangers (RS) study, in which I, alongside Carol Smart, explored the experience of people becoming parents and grandparents through donor conception (for an in‐depth account of these studies and their methodologies, see Nordqvist 2012 and Nordqvist and Smart 2014). These studies, while not drawing on data with donors themselves (predominantly), inquired directly into personal life issues. In doing so, they provide instructive examples of situations in which donors necessarily find themselves, as well as, in some instances, insights into the experiences of donors’ families. Taken together, these sets of studies serve to demonstrate the relevance of a sociology of personal life in the context of donation.

## Donating in context and research

Whereas the first recorded sperm donor insemination was performed in 1884 in the US, the first oocyte donation took place in 1983 in Australia (Harper *et al*. 2016). A number of techniques now enable sperm to be washed, stored, analysed and cryopreserved, but egg freezing remains a challenge with most egg donation procedures requiring eggs to be freshly donated, rather than frozen. In 2013, in the UK alone, over 2000 children were born as a result of egg, sperm or embryo donation (Human Fertilisation and Embryology Authority 2017); by 2014, over 35,000 children had been born in this way in the UK since official records began in 1991 (Nordqvist and Smart 2014).

Donation practices, and the provision of different types of gamete donation, vary within different social and regulatory contexts. The arrangements through which donors donate also differ within and across nations. The form of donor conception most commonly referred to in the literature is when donors donate to someone unknown, usually through the framework of a reproductive health centre. These donors are anonymous to the recipients at the time of the donation, but may donate under an “identity release” framework (as has been the case in the UK since 2005). But there are also more hidden groups of donors that are important to consider, namely, donors who enter into more personal or informal donation agreements with intended parents (e.g. Nordqvist 2011). In such arrangements, donors and prospective parents negotiate for themselves the relationship that exists among the recipient parents, the donor and the child/ren (e.g. Jadva *et al*. 2015, Nordqvist 2011).

There exists a relatively longstanding body of research into recipients of donor gametes, exploring issues associated with forming families in this way (e.g. Freeman *et al*. 2014, Golombok 2015, Melhuus 2012, Nordqvist 2014b). In comparison, as already mentioned, donors have received much less attention. The research that does exist has tended to be quantitative in nature, exploring mainly anonymous donors’ motivations for donating, as well as their attitudes towards donor offspring and potential future contact with said offspring (Crawshaw *et al*. 2007, Daniels and Lewis 1996, Daniels *et al*. 2011, 2012, Freeman *et al*. 2016, Jadva *et al*. 2011, Kirkman *et al*. 2014, Speirs 2012, Wheatley 2017; for a systematic review, see van den Broeck *et al*. 2013). However, as pointed out by van den Broeck *et al*. (2013), donors have not been regarded as people in their own right. Very recently, however, a small body of research has emerged that more fully explores donors’ own experiences and understandings (e.g. Almeling 2011, Goedeke and Daniels 2017, Goedeke *et al*. 2015, Konrad 2005, Mohr 2016, 2018, Shaw 2007).

This tendency is also echoed in the literature on disclosure in this field. Whereas there is research exploring how parents have coped within the recent shift towards greater openness, and their decisions about secrecy versus disclosure (e.g. Golombok 2015, Nordqvist and Smart 2014), we still know very little about how donors navigate this trend. From some small‐scale studies of donors, we do know, however, that donating raises significant issues for donors with regard to their own families: they think carefully about telling their own family members about having donated an egg or sperm. In a US‐based study that mapped rates of disclosure among (mainly sperm) donors, Jadva *et al*. (2011) found that such donors may tell their partners, but are far more reluctant to tell their own parents or children. Daniels *et al*. (2005) found in a small Sweden‐based study that whereas most of the donors (90%) had told their partners, only a third had told their families of origin, thus indicating that information is unevenly shared in families. This study found that close kin are key actors in shaping how donors think about potential future contact with donor offspring, and that donors often feel hesitant about sharing information with said kin in the first place. Indeed, perhaps with good reason, as the same study showed that family members may respond to the news in various ways, and not necessarily well (Daniels *et al*. 2005: 17). This finding was partially echoed by Beeson *et al*. (2013), the only study to date, to my knowledge, to examine how parents of donors view the donation. Crawshaw *et al*. (2007), Daniels *et al*. (2012) and Speirs (2012), exploring anonymous (sperm) donors’ attitudes towards contact with future offspring, suggest that donors may feel a degree of curiosity regarding their donor offspring, but have concerns about the possible detrimental effects of disclosure on their existing family relationships.

## Donating, connectedness and personal life

Relationships matter deeply to people and are hugely significant (e.g. Finch and Mason 2000, Smart 2007). Recognising the importance of a donor's personal life means exploring how donors are connected to other people, i.e. how they are embedded in networks of relationships that are significant, interconnected and enduring (whether positive or negative): a donor's actions, thoughts and decisions are linked to and shaped by their relationships, while the latter are in turn altered by the former.

Relationships with a partner, family or kin are likely to be especially meaningful in this context. There are several reasons for this. First, sociological studies of kinship (e.g. Mason 2008, Nordqvist 2014b) indicate the ongoing importance of intergenerational relationships in personal life, emphasising that vertical and horizontal kin relationships matter. Bengtson *et al*. (2002) note that such relationships are particularly heightened in the context of reproduction because significant others, especially close relatives, constitute important relationships here. Notwithstanding that the reproductive practices considered may be unusual, kin relationships are likely triggered. Bengtson *et al*. (2002, in Smart 2007), suggest that in order to grasp the life of an individual, one must also grasp the lives that it crosses over with, impedes or is impeded by, or that run in parallel to it. As lives are interwoven and embedded in networks, relationships cannot simply end. In Smart's (2007) words, there is a “sticky” quality to these relationships.

The “sticky” and unending quality of family relationships interlinks with the perceived centrality of genetics, biology and blood in Euro‐American cultures (e.g. Nordqvist 2017). A core aspect of these “bodily” concepts of kinship connection is that a biological and/or genetic relationship “fixes” relationships, making them seem in “no sense elective” (Mason 2008). Genetic material is culturally perceived to “connect people together,” and shapes ideas about who is perceived to belong to a family (or not) (Edwards and Strathern 2000). A family (construed in its broadest sense) is “a one” because the individuals within it are perceived to share the “family body”; a “body” that is culturally seen to emerge as genes are passed on from one generation to the next, creating bodily continuity and traceability between generations (Marre and Bestard 2009). As such, the decision to donate has the potential to raise questions within family networks (as opposed to, e.g. networks of friends) because the donor is donating *genetic* material, that is to say, material that is potentially perceived to “belong” to the family.

Genetic relationships are not, however, straightforward in their meaning, definition or application. Studies of what being related means to people in everyday life have shown that there is no clear answer to what genetic links “are” to people, or what relationships they cultivate (e.g. Finch and Mason 1993, Nordqvist and Smart 2014). In contrast to the popular understanding that genetic links between relatives are self‐evidently “there,” empirical sociological and anthropological work suggests that, in everyday life, genetic connections are something “lived,” “felt” and “managed” in complex ways (Mason 2008, Nordqvist and Smart 2014). In a similar vein to Morgan (1996), who argues that family is not something you “are” but something you “do” – “a set of activities” (Morgan 2011: 6) – I see genetic connectedness not as something we “are” but as something we “do”: the living, feeling and managing of genetic connections is also what produces them as real (Nordqvist forthcoming).

A personal life perspective does, however, shift the gaze from exploring “just” genes and kinship, and serves to reveal connectedness as a complex, nuanced, multilayered phenomenon. In the following, therefore, I consider three particular “instances” of connectedness: starting with connectedness as genetic relatedness, then connectedness as relationality and, finally, connectedness and the intimate body.

### “Living” genetic connectedness

One prominent aspect of “living” genetic connections is that such connections inform how people conceptualise their relationships to other people. This point is well illustrated in the following empirical example from the *RS* study, in which Theresa took part, a grandmother of two children born to a lesbian couple who were themselves recipients of donor sperm. Theresa's daughter, the birth mother of the two children, had gone on to donate eggs, making Theresa the mother of an egg donor. Theresa said:

[My daughter] said “I had IVF I donated my eggs.” It was like a bang in my head. Why? Because it is a part of her it is a part of me, it's family. […] A real egg is something from yourself you're giving to somebody you don't know. You don't know how it is going to be brought up. […] I said [to my daughter] “It is you, your genes are in it. It's yours [it] can be [children that] look like you.” […] It's horrible. I dream about it. […] I said “oh my God there are some little children I am the grandmother and I even don't know.” That feels very hard. (*RS* study, lesbian sperm donation)

Biogenetic links are perceived to connect people, not just the donor and the donor‐conceived child, but whole networks of people. Thus, the genetic connection inherent in a donation is not confined to the donor–child dyad, but “cascades” through family networks and may include anyone who reckons themselves family of the donor – like Theresa. The gene operates as a “conduit of relatedness” (Nordqvist and Smart 2014), meaning that everyone in a family network may feel themselves to have a stake in the donation. In other words, relationships here have the ability to mutate quite substantially; they are what Konrad (2005: 49, 173) called “transilient relationships.” This quality fundamentally underscores how relationships are understood. Theresa, it would appear, understands herself to be a grandmother to any genetic offspring of her daughter. These findings were echoed in Beeson *et al*.'s (2013: 1304) US‐based study where 38 per cent of the parents of donors who took part agreed that their initial thought upon finding out about their adult child being a donor was that they “felt happy they might have grandchildren.”^1^

Theresa's account is also instructive as to how genetic thinking is integral to the process of “claiming” relationships, another aspect of “living” genetic connectedness. I refer here to the practice of “claiming” someone as “one's own,” as in “she is one of us” (Edwards and Strathern 2000, Melhuus 2012). Central to Theresa's account (above) is the idea that, culturally speaking, a genetic connection enables claims to be made *to a relationship* (Edwards and Strathern 2000). This transcends geographical contexts as demonstrated by Goedeke and Daniels's (2017) study of embryo donors, and Shaw's (2007) study of egg donors, both of which apply in the context of New Zealand where notions of genetic connectedness or “whakapapa” are culturally central. Both studies show that donors see the eggs/embryos as inalienable from themselves; they take the genetic connection to mean that they will always continue to have a claim on a relationship with the child. Unlike in the UK, donors in New Zealand are able to initiate contact with the donor offspring, if offspring have given their permission and are over the age of 18 (New Zealand Government 2018).

A third process embedded in “living” genetic connectedness relates to the issue of responsibility and the perception – culturally, socially and legally speaking – that one has a responsibility towards one's (genetic) kin (e.g. Finch 1985). Konrad's (2005: 100f.) study of UK‐based egg donors and Goedeke *et al*.'s (2015) New Zealand‐based study suggest that donors are alert to such issues: both studies found that donors experience a moral responsibility to any child conceived using their eggs. Theresa's account suggests that donors’ relatives may feel similarly alive to this issue (see also Beeson *et al*. 2013). The fact that genetic connectedness is so central to the perception that there is a relationship raises further questions, for example, whether there is a gendered dimension between the connections generated by donated egg or sperm, whether grandparents may see themselves as entitled to (genetic) grandchildren, or indeed, how non‐genetic or “mixed” genetic/non‐genetic family groups navigate relationships and responsibilities (see Nordqvist 2015).

The fact that families are made up of a series of individuals who are connected but not identical is important here. Families are bound together by their relationships, but people within them were born in different eras, have lived their formative years in different historical times, and may differ in terms of gender, class, race or educational attainment. The interplay between change and continuity across historical time, alongside new practices such as donating, needs to be navigated (Bertaux and Thompson 2007). A donor may view her responsibility to her donor offspring very differently from her daughter, parent or grandparent. This is something that came to the fore quite powerfully in Theresa's account, where she and her daughter had fallen out over the donation. Such tensions are then likely to become part of how families negotiate their coexistence within interconnected webs of relationships (Smart 2007).

The nuances embedded in “living” genetic relationships tie in, more broadly, with issues of how family relationships are woven around themes of time and space (Morgan 1996: 153). Lingering first on the issue of temporality, the “past” figures, culturally speaking, in family life through the trope of the distant ancestor, echoed in ideas of genetic relationships as permanently “there.” In donating, in terms of the passing of time, what becomes most evident is not so much the past but the future, as well as the issue of descendants. Goedeke *et al*. (2015: 2345) demonstrate that New Zealand embryo donors (and recipients) understand the donation arrangement to give rise to an ongoing and permanent connection, to the extent that one donor in their study considered himself to have a financial responsibility towards the offspring, should anything happen to their parents. This sentiment was echoed in the UK *RS* study where I found that many parents and grandparents saw donors as having “an absent presence” in the family (Nordqvist and Smart 2014).

The future raises important questions about how relationships evolve over time. Family boundaries can require careful management for those involved. Consider the following account of Holly, who became a mother through finding a known egg donor in her friend Gina:

I was a bit concerned that [Gina] had told her son that these boys [her son and my son] were brothers. And I said [to her], “Oh, I hadn't quite seen it like that really,” […] We talked it through and she'd obviously thought it through afterwards and said, “Yes, you're right, they're not.” Because her youngest child has a different father, she divorced with her first husband and she said to me, “Our child is as much a brother to [my eldest children] as their youngest brother […].” And I said, “Well, […] there's a social relationship too, you know. Do you then feel that you're [my son's] mother?” (*RS* study, heterosexual egg donation)

The significance of the genetic relationship cannot just be disregarded, particularly in known donor arrangements and especially with regards to the issue of “donor siblings,” i.e. people who share the same donor. The lack of clarity means that donors and recipients, and their families, may have divergent interpretations of what the relationship *is* and what it *should be*. Taking inspiration from Mary Douglas (1966), donating pushes family relationships into unfamiliar territories and gives rise to an inherently untidy experience of “family.” Perhaps the donor, Gina (in the above quote), felt rather relaxed about the ways in which her genetic offspring related to one another; in contrast, Holly felt acutely that a “relational order” needed to be imposed around her family. This emphasis on managing family boundaries is echoed in studies including donors, with donors showing a strong awareness of the need to not overstep boundaries (e.g. Goedeke *et al*. 2015).

The “transilient” nature of donating becomes crucial here because the people involved in drawing these boundaries may expand over time: Gina and Holly's sons, as genetic “half siblings,” may well develop their own view of the donation as time goes on. The mothers may currently be in a position where they can control whether or not their boys understand themselves to be brothers. That may change considerably, however, as the children grow up and form their own views. Indeed, there is evidence to suggest that donor‐conceived offspring may develop a strong curiosity about their donor siblings and donor (Jadva *et al*. 2010).

The role of physical space in structuring relationships also becomes important in the context of creating relational boundaries to manage these un/familiar connections. Morgan (1996: 139) shows that space is integral to the production and reproduction of relationships: asking questions about space means asking questions about how relational boundaries are drawn. Following Seymour (2007) and Heath *et al*. (2017), the spatial and the social are deeply entwined: space creates, produces and reproduces relationships. Holly, who was anxious about the potential closeness between her own family and that of Gina, took comfort in the fact that Gina lived far away and that the children attended different schools. Through spatial distance, the familial relationship could be preserved, guarded and contained. If donating as a practice threatened to exceed or threaten cherished familial boundaries, so crucial to the social order, then management of space could help to contain this risk. It should be noted that the possibility of managing relational boundaries through geographical distance may be changing with increased Internet usage, and social networking applications such as Facebook and Instagram, which enable new connections to be made to an unprecedented degree and with greater speed than ever before (Chambers 2013). Indeed, the Internet has already enabled the emergence of an online platform that connects donor and donor siblings, namely, the Donor Sibling Registry (DSR) (e.g. Jadva *et al*. 2010). This is not to say that geographical space no longer structures relationships, but that analyses thereof will have to consider the impact on enhanced online connectivity (Andreassen 2018).

### Relationality

Another way in which connectedness comes into play in terms of how people relate to one another is in the everyday. This might seem at first a rather obvious point to make; however, it is important to note just how deeply intimate relationships matter to people, as well as the immense complexity, layers of meaning, history, biography and emotionality that reside within them (e.g. Finch and Mason 1993). In order to grasp the impact of donating on donors’ everyday lives, it is therefore also imperative to grasp the dynamics of ways of relating – “the relationality” (Finch and Mason 2000) – that characterise donors’ personal lives.

Relationality enters into the donating experience on several levels, one being the donor's imagined or real relationship with the recipients. There is evidence to suggest that relationality and the giving of a gift are intimately linked. For instance, Shaw (2007) shows that for egg donors in New Zealand, where donor anonymity is prohibited, part of the process of giving eggs is about establishing some kind of a social bond: the gift is a symbolic gesture imbued with meanings of human solidarity . For many of the donors in Shaw's study, this meant that there was a desire to establish some kind of relationship with the recipients. This is highlighted with particular clarity in one case where a donor found that the recipients had no interest in establishing any kind of bond or relationship with her, leading her to feel “used and abused” (Shaw 2007: 306). In the end, she regretted having donated in the first place. This also signals more broadly that recipients can be highly significant “others” to donors within this process.

Relationality also enters the process on the level of donors’ managing and negotiating their own everyday relationships. In the *RS* study, we interviewed Laura and Natalie, a lesbian couple who had a son through a known sperm donor. The donor was not a friend or family member, but someone they found over the Internet: he was not particularly well known to them, but he was also not completely unknown. He had agreed to donate so that the couple could have two children. However, after their first child was born, he met a new partner, which complicated matters somewhat. Laura said:

[The donor] then got a girlfriend and they became very serious, he didn't want to donate anymore because, yeah, his girlfriend didn't know about it. He was having to, sort of, lie to her, basically. So he said, “Listen, I don't want to carry on with this.” So he's left samples at [a clinic] for us which are now due to be released [so that we can try for a sibling]. (lesbian sperm donation)

To outside observers, Laura and Natalie's donor might be deemed to be acting unwisely and certainly untruthfully. However, such sentiments fail to capture the complexity embedded in personal relationships. The theoretical framework of relationality brings into view how personal relationships emerge and evolve through subtle layers of meaning. Although some relationships, such as those between partners (Heaphy *et al*. 2013), or parents and grandparents (Mason *et al*. 2007, Nordqvist 2015), might be said to come with particular sets of cultural expectations, such expectations do not easily translate into practice. Rather, individuals in couples and families negotiate with each other in a context with no fixed rules, but rather a mixture of hopes and aspirations, habituated modes of conduct, broad principles of relating, personal grief or need, in addition to the impact of the wider cultural and social milieu. For Laura's donor, we can only assume that telling the truth was simply felt to be too challenging for his new relationship. Of course, we do not know why this was the case, but his intention to carefully guard this secret highlights how the act of donating can be experienced as an extremely sensitive piece of information to share with others (see also Speirs 2012).

This situation is echoed in studies with donors, which suggest that donating can trigger questions for partner relationships. For example, Daniels *et al*. (2012: 674), in a study of donors participating in the DSR, record one donor saying that contact with the donor family raised questions at home: “I learnt that my wife had feelings of jealousy when I would spend time online chatting with my donor child's mother.” Along similar lines, Johnson (2017), which also studies donation in the US context, where there is a lack of overarching regulation, found that egg donors’ partners may feel themselves to have a claim to the donor eggs.

In order to account for the experience of donating, it is therefore vital to appreciate the nature of relationships, and specifically the nature of those in which donors are embedded. Donors may feel positively about having donated, but nevertheless remain cautious about telling others. If they do tell others, they might find that a parent (like Theresa, above) or a partner disagrees. Family members play a integral role in personal life, and crucially, because these relationships are “sticky,” they cannot easily be left behind or ignored if their response is unfavourable or judgemental. This means that decisions about disclosure are likely to be significant in donors’ lives.

### The intimate body

I use the term “intimate body” to refer to embodied sexuality and intimacy, seeing them as core parts of personal life, and fundamental to relationships between partners (Gabb and Fink 2015). I use the concept “sexuality” to mainly refer to bodily sexuality and sexual practice, for example, as expressed in masturbation or having sex. But I also acknowledge the relationship between sexual acts and constructions of a sexual self, or sexual identity. I use the term “intimacy” to refer to bodily intimacy, but I also recognise the slippage between the sexual intimate life that one might share with a partner, and the broader use of “intimacy” that refers to intimate relationships in a wider, and not necessarily sexual, sense (e.g. Jamieson 1998). The sexual and intimate aspects embedded in donating are integral to the practice, and yet these aspects have received very little attention in the literature (cf. Mohr 2016, Nordqvist 2011).

The process of donating transposes acts that are culturally considered sexual and private into the semi‐public domain. Emily and her partner Poppy tried to conceive with a named but uninvolved donor who, after some months of donating, moved to live into shared housing. The couple used to go and visit at the time of ovulation to retrieve a donation, but the communal living arrangements became trying:

We went there; we sat round the table; and then he has to go upstairs and they're [the others living there] all going “yay, go on, go on.” And it's like this is just … you know, it's losing any sense of comfortable … I don't know … it just felt weird, you know? Then he comes down all red faced and then they all take the mick out of him and then we have to go upstairs and everybody knows exactly what you're doing, you know, in a kind of literal sense, not in a general sense. You know “oh, yes, they're trying for a baby.” You know, “now I know exactly what you're doing upstairs with that yoghurt pot.” (Emily, *CT* study, lesbian sperm donation)

Emily's account is suggestive of the strong feelings of discomfort associated with crossing intimate and sexual boundaries. Following Emily's account, what was disturbing to her was not just the need to be open about attempting to conceive, but also needing to make explicit the physical and material dimensions embedded within that process. Of course, not all donations will take place in the semi‐public view of a communal household, but every donation will require that donors – and recipients (Nordqvist 2011, 2012) – to an extent manage their sexual and intimate bodies in a public or semi‐public setting, be that in the context of a reproductive health centre, or in the case of sperm donors, in a private arrangement. This is fraught because embodied sexuality and intimacy are so strongly associated with spatial realms that are defined as private, such as the home (Gabb and Fink 2015) and specific areas of the home (e.g. Gabb 2013).

Mohr's (2016) research on donors’ experiences offers ample evidence that donors too are sensitive to these issues. He shows how donors can feel acutely awkward about entering into the space of a sperm bank; one donor describes the atmosphere of a sperm bank waiting room as one of anxiety and male shame: “whatever happens here is private” (Mohr 2016: 331). Semen, bringing with it the potential of both lust and disgust, requires careful management in the space of the bank: Mohr (2016) suggests that banks deploy material‐semiotic practices aimed at making sperm into a “governable” substance.

Intimacy and sexuality also come into play because of how the intimate body is necessarily engaged in donating. The following two extracts are from the website of British sperm and egg banks as they seek to recruit sperm and egg donors. The aim of the content is to describe the process of donating to prospective donors:

As a sperm donor, you can visit the clinic to make donations any time during working hours (8.30 am–3.30 pm Monday–Friday). […] You will be asked to donate once or twice a week for a period of 3–6 months. […] A commitment to making regular donations is important. You will need to ensure you have 3 days abstinence between donations so you can provide the best sample possible. (London Sperm Bank 2018)

Sperm donors can only donate during office hours, so it is likely that they will need to manage donating among work responsibilities. The website emphasises the need for frequency, so it is clear that this is something sperm donors need to manage on a regular basis, over the course of weeks and months. They also need to be abstinent for 3 days before donating. If donating twice weekly, that could leave very little room for having a sexually active life of their own. This raises questions about the potential impact of donating on their own intimate lives, and their life with a partner, especially if they are donating in secret.

Whereas sperm can be frozen, eggs are usually donated “fresh,” and egg donors are required to synchronise their cycle with the recipient woman. According to the website of the London Egg Bank, this means that the egg retrieval happens in stages. It states that as part of the final stage:

We will […] arrange for you to come in for a baseline transvaginal ultrasound scan on day 2 or 3 of your period. The scan will visualise the status of your ovaries and provide a guide to when medication for ovarian stimulation should be started. (London Egg Bank 2018)

Drawing on Emerson (1970), considerable effort is required of doctors and women throughout gynaecological investigations in order for reality to be sustained so that the process may be defined as non‐intimate. This aspect of intimacy in egg donation has received rather less attention than other bodily dimensions embedded in egg donation, including taking hormonal drugs and surgery (e.g. Almeling 2011, Nahman 2013).

The bodily dimensions of donating require considerable management of the intimate body, an area of life often regarded as very private, if not secret. This is likely to compound the felt impact of these issues. According to a recent survey of Danish donors, where donors can choose whether to remain anonymous, approximately half of the respondents had either never discussed being a donor, or had only talked about it with a select few (Bay *et al*. 2014). The felt need for secrecy is perhaps most likely to be an issue in sperm donation, linking back to its sexual dimensions. Historically and religiously, masturbation was once deemed an act indicative of an innate sexual perversion (Hawkes 1996). Foucault (1990 [1979]) has shown that during the 19th century, sexuality became deeply linked to the production of moral subjectivity and identity; masturbation was seen to corrupt the whole moral being of a young man. Although such ideas may have diminished somewhat, they remain deeply ingrained in many respects. An example of how such views persist in today's lexicon is the derogatory English term “wanker,” which, according to the Oxford English Dictionary (2017), means “one who masturbates” and also “an objectionable or contemptible person,” denoting its strong and negative moral connotations. In other words, male masturbation is associated with moral disgrace and shame. Men who donate are likely to need to manage the negative associations that accompany sperm donation.

## Conclusions

In this article, I have shown how issues of personal life feature in donating, and I have suggested that a sociology of personal life can usefully be put to work to capture something vital about the donating experience that remains under‐researched. In conclusion, I want to offer some thoughts about how tuning in to these issues might contribute to a shift in the field at large.

First of all, an exploration of connectedness as a multidimensional quality in this context sheds light on new aspects of donation, that is, as a decision and event with likely implications for donors’ own everyday lives and relationships. If researchers are to properly capture the process through which this happens, their projects need to move beyond “surface mapping” donors’ behaviours and attitudes, and begin to incorporate the various ways in which donating is situated within people's lives. An approach that aims to investigate the relational dynamics that underpin donors’ practices and understandings, and that seeks to better understand the potential sensitivities associated with donating, arguably calls for a shift from quantitative, survey‐based research designs to more qualitatively driven pieces of research.

Second, a sociology of personal life proposes that a sense of meaningful connectedness can be found in both likely and unlikely places. To this end, Törnqvist (2018) argues for the need to explore intimate life as something that is not confined to a set of relationships, but rather as a situational quality: as a specific mode of interaction and particular experience of closeness. Such an approach suggests that researchers need to take a “bottom‐up approach” and explore open‐endedly how connectedness might matter to donors, rather than making assumptions about donors’ experiences. Within the current field of policy‐making and research on donors’ attitudes towards their donor offspring (e.g. Crawshaw *et al*. 2007, Daniels *et al*. 2012, Jadva *et al*. 2011, Speirs 2012, Wheatley 2017), there is a widespread (and understandable) assumption that *this* form of connection is what really matters (for a critical engagement with such understandings, see Gilman and Nordqvist 2018). If the act of donating is a way of forming social bonds, as suggested by Shaw (2007), it is, I think, an empirical question as to how that plays out. Further research is needed to explore how and with whom donors experience meaningful forms of connectedness. Might it include, for example, recipients, other donors or even clinic staff?

Third, a personal life perspective considers donors as individuals who live connected lives, and as a result, new questions emerge about how donating has an impact on donors’ relational worlds. How do donors experience the disclosing of information to their own families, and how does that process affect relationships? Might people within the same family view the “transilient” relationships that stem from the donation quite differently, and if so, how might that impact on relational networks? In terms of sexual identity, how do donors (especially men) navigate disclosure, while also preserving their own sense of moral integrity or identity; how do they tell their “story” about being a donor? How do donors manage their intimate body in the context of it both being engaged and coming into semi‐public view? Moreover, the framework of personal life can uncover the ways in which related developments in reproductive medicine, such as “social” egg freezing, with its own temporal and relational dynamics, may also have an impact on personal lives. Moreover, in the background to all of these questions, there is the issue of how a donor's sexuality, class, “race”/ethnicity and gender, among other things, interact and intersect to inform experiences.

In my view, such questions signal the need for a new research agenda in respect of donating because, arguably, we cannot fully account for it as a social phenomenon unless we become more attuned to how donating impacts on donors’ own lives and relationships.

## Note

1 This was a quantitative study, and so the word “grandparent” was chosen for participants rather than it being their own word. Nevertheless, it is noteworthy that a large proportion of the participants agreed with this statement.
